# Amyotrophic Lateral Sclerosis in a Patient Treated for Neurosyphilis: A Case Report

**DOI:** 10.3390/neurolint18100193

**Published:** 2026-10-15

**Authors:** Maciej Giecko, Filip Bossowski, Magdalena Bossowska, Anna Moniuszko-Malinowska, Monika Chorąży, Magdalena Sarnowska, Sambor Grygorczuk

**Affiliations:** 1 Department of Infectious Diseases and Neuroinfections, Medical University of Białystok, Żurawia 14, 15-540 Białystok, Poland; maciej.giecko@sd.umb.edu.pl (M.G.);; 2 Student Research Group, Department of Infectious Diseases and Neuroinfections, Medical University of Białystok, Jana Kilińskiego 1, 15-089 Białystok, Poland; 3 Department of Neurology, Medical University of Białystok, Curie-Skłodowskiej 24A, 15-276 Białystok, Poland

**Keywords:** amyotrophic lateral sclerosis, neurosyphilis, *Treponema pallidum*, electromyography, nerve conduction studies, syphilitic amyotrophy, ALS mimic, case report

## Abstract

Background/Objectives: Neurosyphilis is a recognized treatable mimic of amyotrophic lateral sclerosis (ALS); syphilis serology is commonly included in the work-up of suspected motor neuron disease. We describe the converse situation: neurosyphilis that delayed recognition of coexisting ALS. Methods: A 46-year-old man with type 2 diabetes mellitus and degenerative spine disease presented with a six-month history of progressive limb weakness and gait disturbance. Serum syphilis serology was reactive (Venereal Disease Research Laboratory [VDRL] titer 1:32; *Treponema pallidum* hemagglutination assay [TPHA] titer 1:20,480), and cerebrospinal fluid (CSF) showed a mild mononuclear pleocytosis with raised protein and immunoglobulin G. Neurosyphilis was diagnosed and treated with a 14-day course of intravenous penicillin. The subsequent course was followed with neuroimaging and electrodiagnostic studies. Results: Despite treatment and resolution of the CSF pleocytosis, weakness and wasting progressed and bulbar involvement developed. Examination showed combined upper and lower motor neuron signs, electromyography showed an anterior horn cell pattern with widespread fasciculations, and cervical spine imaging showed degenerative changes without spinal cord compression. ALS was diagnosed at a later neurological admission. Conclusions: Identifying a treatable mimic does not close the diagnostic process. Combined upper and lower motor neuron signs that continue to progress despite adequate treatment of neurosyphilis should prompt early electrodiagnostic evaluation for ALS, even though the relative contribution of each condition may remain uncertain.

## 1. Introduction

Syphilis is a systemic bacterial infection caused by the spirochete *Treponema pallidum* [1]. The disease is characterized by a complex clinical course and may progress through several stages if left untreated: primary, secondary, latent, and tertiary [2]. Due to its ability to present with a wide range of symptoms, syphilis is often referred to as “the great imitator,” as it may mimic numerous other diseases [3]. If left untreated, syphilis may lead to severe complications, including involvement of the cardiovascular and nervous systems [3].

When the central nervous system becomes affected, the condition is referred to as neurosyphilis. According to reports by Hamill and colleagues, up to 30% of patients may develop nervous system involvement during the early stages of *Treponema pallidum* infection, sometimes within days after exposure to the bacterium [4]. Neurosyphilis may occur at any stage of the disease, including primary, secondary, latent, or tertiary syphilis, and may develop even in the absence of classical symptoms such as chancres, rash, or mucosal lesions [2].

Traditionally, the clinical spectrum of neurosyphilis has been associated with late stages of untreated infection and may include severe neurological complications such as general paresis or tabes dorsalis [5]. However, contemporary clinical experience shows that neurosyphilis may present with a wide range of neurological manifestations, including headaches, cranial nerve palsies, ataxia, psychiatric disturbances, depression, stroke, epilepsy, and sensory abnormalities [1,2]. This broad spectrum of symptoms may significantly complicate the diagnostic process, particularly in patients with coexisting conditions such as human immunodeficiency virus (HIV) infection, in whom the immune response to *Treponema pallidum* may be altered [3,6]. Motor presentations include syphilitic amyotrophy and syphilitic meningomyelitis, which can produce combined upper and lower motor neuron signs and are recognized mimics of amyotrophic lateral sclerosis (ALS) [7].

ALS is a progressive neurodegenerative disorder characterized by degeneration of upper and lower motor neurons, leading to progressive muscle weakness, muscle atrophy, fasciculations, and spasticity. The etiology of ALS remains incompletely understood. Proposed risk factors include genetic predisposition, exposure to environmental toxins such as heavy metals, repeated head trauma, and smoking, whereas oxidative stress and neuroinflammation are implicated in its pathogenesis [8,9].

The disease is characterized by a steadily progressive course without periods of remission. Most patients develop severe motor disability, and death usually occurs within three to five years after symptom onset, most commonly due to respiratory muscle failure [8]. Because neurosyphilis is a treatable mimic, syphilis serology is commonly included in the work-up of suspected ALS. We describe the converse situation: a patient diagnosed with neurosyphilis in whom a progressive upper and lower motor neuron syndrome, initially attributed to the infection, was diagnosed as ALS, and we discuss the clinical, laboratory, and electrodiagnostic features that help to separate the two conditions.

## 2. Case Presentation

A 46-year-old man with a medical history of hypertension, type 2 diabetes mellitus, hyperlipidemia, and degenerative spine disease developed progressive muscle weakness affecting both the upper and lower limbs over a period of six months. The weakness was accompanied by significant gait disturbances and gradually worsened, eventually limiting the patient’s ability to perform daily activities. Marked muscle atrophy of the upper limbs was observed, particularly in the left arm, to the extent that the patient was unable to hold a pen. Three months after symptom onset he had been hospitalized in a neurology department at another hospital, where brain magnetic resonance imaging (MRI) showed no focal lesions.

On admission, the patient was hemodynamically stable. His heart rate was 90 beats per minute, and auscultation of the lungs revealed normal vesicular breath sounds. Laboratory testing performed on admission revealed no elevated inflammatory markers and no evidence of HIV infection. Viral hepatitis was excluded. Serological testing demonstrated the presence of antibodies against *Treponema pallidum*, with a reactive *Treponema pallidum* hemagglutination assay (TPHA; titer 1:20,480) and a reactive Venereal Disease Research Laboratory (VDRL) test (titer 1:32). A lumbar puncture was subsequently performed. Cerebrospinal fluid (CSF) was clear and colorless, with 7 cells/µL (all mononuclear), no red blood cells, protein 0.72 g/L (reference 0.15–0.45 g/L), albumin 487.5 mg/L (reference 100–300 mg/L), immunoglobulin G (IgG) 85.1 mg/L (reference 10–30 mg/L), and glucose 3.7 mmol/L; a multiplex polymerase chain reaction (PCR) panel for viral and bacterial pathogens was negative. On the basis of the history, the reactive serological tests, and the inflammatory CSF profile [10], neurosyphilis was diagnosed; retrospectively, the findings meet the current German guideline criteria for probable neurosyphilis [11].

Screening serology for *Borrelia burgdorferi* was reactive for immunoglobulin M (IgM) but non-reactive for IgG antibodies, and confirmatory immunoblot (Western blot) testing was negative. The isolated IgM reactivity was therefore regarded as a false-positive result, which can occur in patients with syphilis because of antigenic cross-reactivity [12]. MRI of the cervical spine was performed to exclude structural causes of the observed muscle weakness and atrophy. It showed multilevel degenerative disk bulges from C3/4 to C6/7 and mild marginal osteophytosis along the anterior and posterolateral vertebral margins with indentation of the dural sac and foraminal narrowing, with possible nerve root compression, but no spinal cord compression and no intramedullary signal change, contrast enhancement, or restricted diffusion; neurosurgical consultation found no compression of neural elements and no indication for surgery.

The patient denied any history of genital lesions typical of syphilis. When questioned regarding potential exposure to sexually transmitted infections, he reported that his last casual sexual contact had occurred approximately ten years earlier. For the previous seven years, his only sexual partner had been his wife. The patient’s wife had undergone routine screening for syphilis during pregnancy four years earlier, which had yielded negative results. He had never been treated for syphilis; approximately one year before admission he had received an antibiotic, probably for tonsillitis.

During hospitalization, the patient underwent dermatological consultation. Further specialist consultations in neurology, cardiology, and ophthalmology were recommended to evaluate possible organ involvement associated with syphilis. Ophthalmological examination revealed no abnormalities; in particular, there were no Argyll Robertson pupils. The neurological consultant recommended electroneurography in addition to treatment of syphilis.

Neurological examination demonstrated fasciculations in the upper arm muscles; wasting of the hand and upper arm muscles, more pronounced on the left; brisk deep tendon reflexes in all four limbs, more pronounced in the lower limbs; bilateral ankle clonus; flexor plantar responses; absence of meningeal signs; and a spastic–ataxic gait. Weakness affected the upper and lower limbs, more markedly on the left. The patient reported no sensory disturbances.

The patient was treated with intravenous penicillin (crystalline benzylpenicillin, 24 million units per day, given as 4 million units every 4 h for 14 days) in accordance with treatment guidelines for neurosyphilis [10,13]. Supportive therapy for neurological symptoms was also provided. During hospitalization, a slight improvement in the patient’s general condition was observed; however, muscle weakness of the upper limbs persisted, with little gain in mobility.

Two months after discharge, the patient was readmitted for evaluation of the treatment outcome. At that time, he reported worsening of his general condition and progressive muscle weakness. Physical examination revealed further progression of muscle atrophy and the presence of fasciculations. The patient also reported an increased tendency to fall. During this hospitalization, imaging studies were repeated. A computed tomography (CT) scan of the head revealed no significant abnormalities. MRI of the cervical spine demonstrated degenerative changes and osteophytic formations but no evidence of focal lesions within the spinal cord. A neurological consultation recommended follow-up imaging within three to six months. A repeat lumbar puncture showed 4 cells/µL and a CSF protein concentration of 0.80 g/L, and serum *Treponema pallidum* antibodies remained reactive (247 U). Left-sided nerve conduction studies and needle electromyography performed during this admission showed absent motor responses on distal stimulation of the median, ulnar, peroneal, and tibial nerves, low-amplitude responses (0.28–1.19 mV) on more proximal stimulation of the ulnar, peroneal, and tibial nerves, and preserved ulnar conduction velocities across the elbow and in the upper arm (49.8 and 54.5 m/s). As absent distal responses are physiologically implausible when proximal stimulation of the same nerve evokes a response, these distal findings most likely reflect a technical recording error and were not interpreted. Needle examination of the biceps femoris, tibialis anterior, and vastus lateralis showed neurogenic motor unit changes with a full interference pattern, and fasciculations in the biceps femoris. The nerve conduction findings were reported as axonal damage. Antibiotic therapy was not resumed, as there was no clear indication for retreatment.

The patient subsequently underwent inpatient neurological rehabilitation, completed approximately five months after the first admission, by which time he had become dependent on a wheelchair. Due to persistent neurological deterioration and lack of clinical improvement, he was then admitted to the Department of Neurology for further evaluation. Neurological examination revealed signs consistent with bulbar syndrome, including dysphagia. Dysphagia was a presenting complaint, and on neuropsychological assessment speech was fully intelligible but with reduced voice sonority and flattened intonation. The patient also presented with quadriparesis with features of lower motor neuron involvement, including muscle atrophy and fasciculations predominantly affecting the upper limbs. In addition, signs of upper motor neuron involvement were observed, including increased muscle tone, hyperreflexia, and clonus in the lower extremities. Laboratory tests showed a serum creatine kinase of 115 IU/L (reference 30–200 IU/L), a normal serum protein electrophoresis pattern, glycated hemoglobin (HbA1c) of 6.4%, total serum vitamin B12 of >128 pmol/L, and normal homocysteine (7.67 µmol/L) and folate (8.7 ng/mL) concentrations.

**Figure 1:**
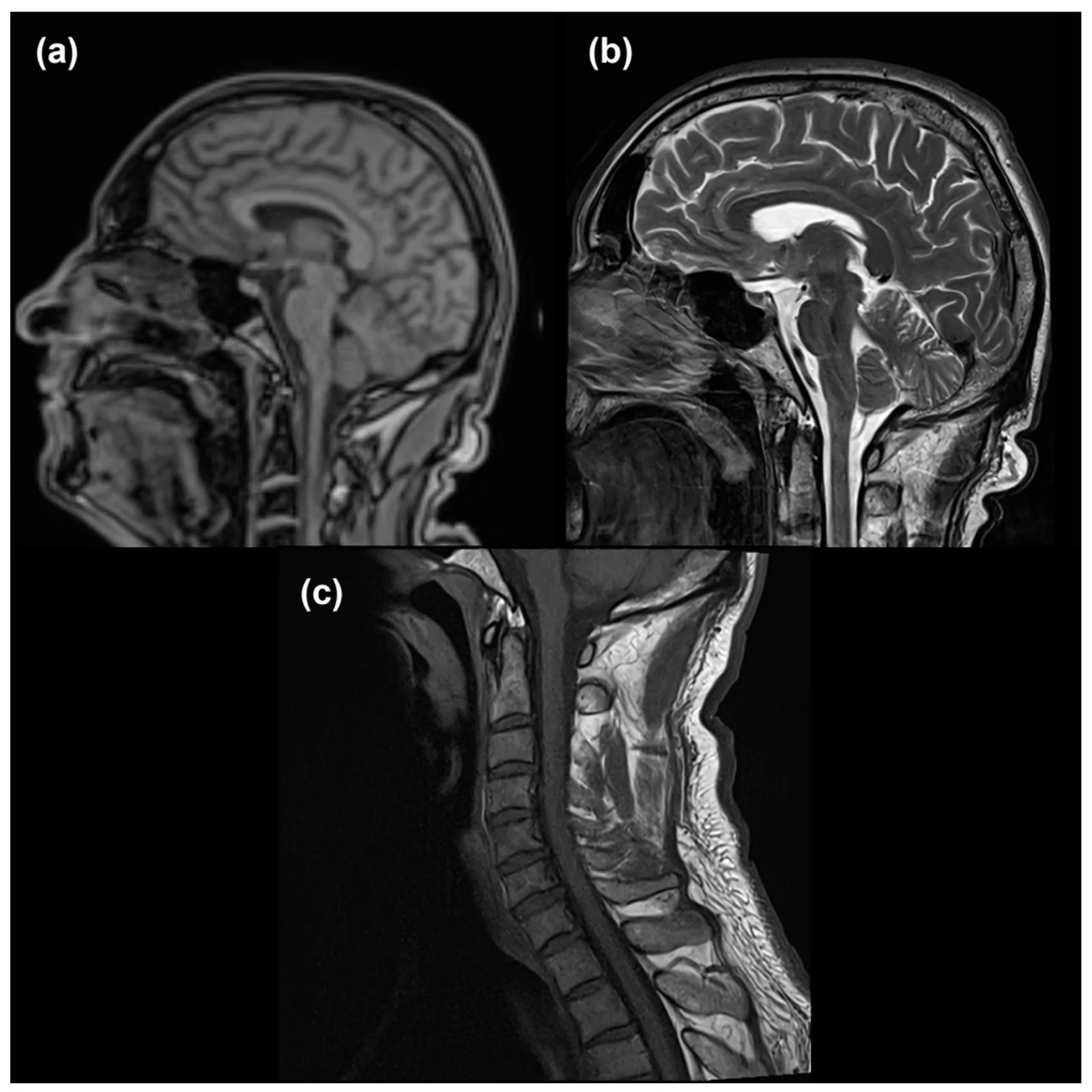
Neuroimaging findings in a patient with amyotrophic lateral sclerosis previously treated for neurosyphilis. Sagittal brain MRI (**a**,**b**) demonstrating mild cortical atrophy without focal intracranial pathology. Sagittal cervical spine MRI (**c**) showing multilevel degenerative spondylotic/disk changes without focal spinal cord lesion.

A cranial CT scan demonstrated a few small periventricular hypodense foci, most likely of vascular origin. MRI of the brain revealed mild cortical atrophy with moderate widening of the adjacent CSF spaces. MRI of the cervical spine—which also covered the upper thoracic spine—showed broad-based disk protrusions at C5/6 and C6/7 indenting the thecal sac, with foraminal narrowing, and a slight right posterolateral disk protrusion at Th2–Th3, without focal spinal cord lesions, pathological enhancement, or restricted diffusion (Figure 1).

Electromyography and nerve conduction studies were subsequently performed. Motor nerve conduction studies revealed markedly reduced amplitudes and prolonged latencies in the right median and ulnar nerves, indicating significant impairment of motor nerve function in the upper limbs. In contrast, conduction parameters in the lower limbs remained relatively preserved (Table 1). The neurography report described the upper-limb responses as trace responses, with distal compound muscle action potentials of 0.55 mV in the median and 0.23 mV in the ulnar nerve. Because conduction block cannot be reliably assessed when the distal response is below 1 mV [14], the reductions in proximal amplitude were not interpreted as conduction block.

At rest, fasciculations were present in all examined muscles. Analysis of motor unit potentials (MUPs) indicated neurogenic changes characterized by significantly increased amplitude and duration, together with a high percentage of polyphasic potentials. Notably, the right biceps, tibialis anterior, and vastus lateralis muscles exhibited mean amplitudes of 1634 μV, 1209 μV, and 2012 μV, respectively, with corresponding durations and polyphasic percentages indicating chronic denervation (Table 2). The interference pattern was reduced, reflecting decreased recruitment, and voluntary activation was absent in the first dorsal interosseous muscle. The electromyographer concluded that the findings indicated motor neuron involvement at the level of the anterior horn cells.

Sensory nerve conduction studies showed reduced conduction velocities and amplitudes in the upper limbs. The right median and ulnar sensory nerves demonstrated prolonged peak latencies and decreased amplitudes (Table 3).

On the basis of the progressive clinical course, the combination of upper and lower motor neuron signs, the bulbar involvement, and the electrodiagnostic findings, a diagnosis of ALS was made (recorded at discharge as provisional, pending observation); the findings fulfill the Gold Coast criteria [15]. The treated neurosyphilis was regarded as a coexisting condition that did not account for the continued progression of the motor syndrome (Table 4). The patient, already dependent on a wheelchair, remained clinically stable during this hospitalization and was given information about ongoing clinical trials in ALS and about patient associations. Riluzole 50 mg twice daily was started at discharge, and he was referred to outpatient neurology, genetics, and pulmonology clinics. He was subsequently lost to follow-up.

## 3. Discussion

In the context of ALS, neurosyphilis is usually considered a treatable mimic, in which identifying the infection resolves the diagnostic problem. The present case illustrates the converse: the mimic was genuinely present, diagnosed, and adequately treated, yet it did not explain the progressive motor syndrome. Once a treatable cause has been identified, diagnostic momentum favors attributing all subsequent findings to it; the lesson of this case is that an established diagnosis should be tested against the clinical course rather than allowed to close the diagnostic process.

In Poland, epidemiological data indicate a gradual increase in the number of reported syphilis cases. In 2019 alone, 1511 cases were reported, representing an increase compared with previous years [16]. As its prevalence rises, syphilis will be encountered more often as a coexisting finding in patients evaluated for other neurological disorders, including motor neuron disease.

In the present case, reactive serum serology in a previously untreated patient, together with a mild mononuclear pleocytosis and raised CSF protein and IgG, led to a diagnosis of neurosyphilis, which was initially considered the cause of the patient’s neurological syndrome. A reactive *Borrelia* IgM, later regarded as a false-positive result, had initially added neuroborreliosis to the differential diagnosis, so that two infectious explanations were under consideration at that stage. Electroneurography was recommended at the neurological consultation during the first admission. When it was performed at the follow-up admission, nerve conduction studies and electromyography already showed low-amplitude motor responses, neurogenic motor unit changes, and fasciculations, but the findings were reported as axonal damage; the anterior horn cell pattern was recognized only when the study was repeated at the neurological admission almost four months later. In retrospect, this anchoring on the infection delayed consideration of a coexisting motor neuron disease; it was the continued progression of the deficits despite treatment, together with the emergence of bulbar involvement, that prompted further neurological evaluation.

The relative contribution of each condition to the patient’s deficits cannot be determined with certainty. A serum VDRL titer of 1:32 in a previously untreated patient indicates active infection rather than past exposure, and treatment of neurosyphilis was clearly indicated. The CSF inflammatory response, however, was modest: the pleocytosis was mild (7 cells/µL), and the raised protein concentration (0.72 g/L) is non-specific and may also be seen in ALS and in diabetes mellitus. At follow-up, the pleocytosis had resolved (4 cells/µL), consistent with a response to treatment [10], whereas the protein concentration remained raised (0.80 g/L), a non-specific finding, and the motor syndrome continued to progress. Persistent neurological deficits after adequate penicillin therapy do not in themselves exclude neurosyphilis, because sequelae may remain despite successful treatment [13]; in this patient, however, it was the continued progression of the deficits over the following months and the emergence of bulbar involvement that argued against neurosyphilis as the sole explanation.

Neurosyphilis is itself a recognized mimic of ALS. Syphilitic amyotrophy and syphilitic meningomyelitis can produce combined upper and lower motor neuron signs, and an ALS mimic syndrome due to neurosyphilis has been reported [7]; a treatable syphilitic cause must therefore be considered in any patient with an ALS-like presentation and reactive serology. Progression despite appropriate treatment should prompt either re-evaluation for treatment failure, including repeat CSF examination [13], or consideration of an alternative diagnosis.

The findings fulfill the Gold Coast criteria [15], which require progressive motor impairment documented by history or repeated clinical assessment; upper and lower motor neuron dysfunction in at least one body region, or lower motor neuron dysfunction in at least two body regions; and investigations excluding other disease processes. The first two requirements were met: progression was documented across repeated examinations, upper and lower motor neuron involvement was present in the lumbosacral region (hyperreflexia, clonus, and increased tone on examination, and fasciculations with chronic neurogenic change in the tibialis anterior and vastus lateralis on electromyography), and both were also present in the cervical region (brisk reflexes, documented at the first admission, in weak and wasted upper limbs, together with fasciculations and no voluntary activation of the first dorsal interosseous), in addition to bulbar involvement. Under these criteria, lower motor neuron dysfunction may be established by clinical examination as well as by electromyography. The third requirement is where the diagnostic difficulty lay, because neurosyphilis is itself a potential mimic, as discussed above.

Multifocal motor neuropathy and chronic inflammatory demyelinating polyneuropathy were considered because of the prolonged distal motor latencies, the reduced ulnar conduction velocity, and the reductions in proximal compared with distal amplitudes in Table 1. Conduction block cannot be reliably assessed when the distal compound muscle action potential is below 1 mV [14], and the distal responses here were 0.55 mV and 0.23 mV, described in the neurography report as trace responses; with responses this small, conduction velocity is also unreliable, because the fastest-conducting axons are preferentially lost and latency measurement becomes imprecise. Multifocal motor neuropathy does not produce upper motor neuron signs, which were prominent in this patient, and chronic inflammatory demyelinating polyneuropathy would be expected to cause hyporeflexia or areflexia rather than the hyperreflexia and clonus observed. In the earlier left-sided study, ulnar conduction velocities across the elbow and in the upper arm were preserved (49.8 and 54.5 m/s), which further argues against a diffuse demyelinating process.

Cervical spondylotic myelopathy and radiculopathy were considered because of the multilevel degenerative changes, dural sac indentation, and foraminal narrowing on imaging. However, there was no spinal cord compression or intramedullary signal change, neurosurgical assessment found no compression of neural elements, and cervical disease would not account for the lower motor neuron involvement of the lower limbs or for the bulbar involvement. The cervical MRI, which extended to the upper thoracic spine, showed only a slight Th2–Th3 disk protrusion without spinal cord compression or signal change, and a thoracic lesion could not in any case explain the brisk reflexes and lower motor neuron signs in the upper limbs, the bulbar involvement, or the denervation of lower limb muscles. The anterior cervical osteophytes were mild, making a mechanical cause of the dysphagia unlikely.

The sensory conduction abnormalities are atypical for ALS, which is a disorder of the motor system. The prolonged distal motor latencies in the median and ulnar nerves (7.96 and 6.98 ms) and the slowed median sensory conduction (27.8 m/s) exceed what axonal loss alone usually produces. A superimposed entrapment neuropathy at the wrist, such as carpal tunnel syndrome, and a coexisting diabetic polyneuropathy may both contribute, whereas the ulnar forearm velocity was derived from a trace proximal response and cannot be interpreted. Diabetic polyneuropathy is less likely given good glycemic control (HbA1c 6.4% on metformin) and the absence of documented diabetic complications, but it cannot be excluded. Neither process would account for the upper motor neuron signs, the bulbar involvement, or the widespread denervation.

Similar diagnostic challenges have been described in previously reported cases of neurosyphilis presenting with atypical neurological manifestations, including a cognitive and psychiatric presentation initially investigated as neuroborreliosis [17]. Neurosyphilis has also presented as suspected encephalitis with dysarthria and central facial palsy [18].

This report has limitations inherent to a single retrospective case: it relies on routine clinical records, and some investigations recommended in the work-up of suspected ALS were not available. A follow-up serum VDRL titer is not documented, so the serological response to treatment could not be assessed. The diagnosis of ALS was recorded as provisional at discharge, and because the patient was subsequently lost to follow-up, it could not be confirmed by the later course. The relative contribution of neurosyphilis and ALS to the patient’s deficits cannot be established with certainty, and a single case cannot establish whether their coexistence was coincidental.

## 4. Conclusions

Identifying a treatable mimic does not close the diagnostic process. Progressive combined upper and lower motor neuron signs, particularly with bulbar involvement, that continue despite adequate treatment of neurosyphilis should prompt evaluation for ALS rather than being attributed to the infection or to treatment failure. Conversely, syphilis should continue to be sought in patients with an ALS-like presentation, because neurosyphilis remains a treatable mimic. When the two conditions coexist, the contribution of each may remain uncertain, and electrodiagnostic evaluation should therefore accompany, rather than follow, antimicrobial treatment.

Early recognition of ALS is crucial for appropriate patient management, initiation of supportive therapy, and planning of long-term care. Although curative treatment for ALS remains unavailable, early diagnosis allows for implementation of multidisciplinary care strategies that may improve quality of life.

## Figures and Tables

**Table 1:** Motor nerve conduction studies (MNCS).

Nerve	Segment	Latency (ms)	Amplitude (mV)	Conduction Velocity (m/s)
Right Median Motor	Wrist to APB	7.96	0.55	
	Elbow to Wrist	19.1	0.03	39.5
Right Ulnar Motor	Wrist to ADM	6.98	0.23	
	Below Elbow to Wrist	17.6	0.015	21.7
Right Peroneal Motor	Ankle to EDB	4.93	3.1	
	Fibular Head to Ankle	12.6	2.9	48.2
Right Tibial Motor	Ankle to Abductor Hallucis	4.1	2.5	
	Mid-Calf to Ankle	13.9	0.98	38.8

APB: Abductor Pollicis Brevis; ADM: Abductor Digiti Minimi; EDB: Extensor Digitorum Brevis.

**Table 2:** Electromyography (EMG) motor unit potential data.

Muscle	Mean Amplitude (μV)	Mean Duration (ms)	% Polyphasic
Right Biceps	1634	15.8	15
Right Tibialis Anterior	1209	18	30
Right Vastus Lateralis	2012	15.3	42.9

**Table 3:** Sensory nerve conduction studies (SNCS).

Nerve	Segment	Peak Latency (ms)	Amplitude (μV)	Conduction Velocity (m/s)
Right Median Sensory	Digit I to Wrist	6.15	10.1	27.8
Right Ulnar Sensory	Digit V to Wrist	5.29	5.3	32.4

**Table 4:** Timeline of clinical events (month 0 = first admission).

Time	Setting	Findings and Management
Months −6 to 0	Outpatient	Progressive weakness of the upper and lower limbs with gait disturbance
Month −3	Neurology department, another hospital	Brain MRI without focal lesions
Month 0 (3 weeks)	First admission, Department of Infectious Diseases and Neuroinfections	Serum VDRL 1:32, TPHA 1:20,480; CSF 7 cells/µL, protein 0.72 g/L, raised immunoglobulin G; diagnosis of neurosyphilis; cervical MRI: degenerative changes without cord compression; fasciculations, wasting, hyperreflexia, ankle clonus; electroneurography recommended; intravenous crystalline benzylpenicillin 24 million units/day for 14 days
Month 2.5	Second admission, Department of Infectious Diseases and Neuroinfections	Progressive weakness and wasting, falls; repeat imaging; repeat CSF 4 cells/µL, protein 0.80 g/L; left-sided nerve conduction study and electromyography, reported as axonal damage; antibiotic therapy not resumed
Up to month 5	Inpatient neurological rehabilitation	Wheelchair dependence
Month 6	Department of Neurology	Bulbar involvement with dysphagia; upper and lower motor neuron signs; electromyography: anterior horn cell pattern with fasciculations in all examined muscles; diagnosis of ALS (provisional); riluzole started
After diagnosis	Follow-up	Lost to follow-up

## Data Availability

The original contributions presented in this study are included in the article. Further inquiries can be directed to the corresponding author.
